# *“I will never wish this pain to even my worst enemy”*: Lived experiences of pain associated with manual vacuum aspiration during post-abortion care in Kenya

**DOI:** 10.1371/journal.pone.0289689

**Published:** 2023-08-24

**Authors:** Ramatou Ouedraogo, Valleria Obure, Grace Kimemia, Anne Achieng, Mercy Kadzo, Jane Shirima, Shilla Unda Dama, Shelmith Wanjiru, Jonna Both

**Affiliations:** 1 African Population and Health Research Center, Nairobi, Kenya; 2 Rutgers, Utrecht, Netherlands; OAUTHC: Obafemi Awolowo University Teaching Hospital Complex, NIGERIA

## Abstract

**Background and objectives:**

In Kenya, where abortion is legally restricted, most abortions are induced using unsafe procedures, and lead to complications treated in public health facilities. The introduction of Manual Vacuum Aspiration (MVA) to treat incomplete abortion has improved the management of abortion complications. However, this technology comes with pain whose management has been a challenge. This paper explores the lived experiences of pain (management) during MVA to document the contributing factors.

**Methods:**

We used an ethnographic approach to explore girls and healthcare providers’ experiences in offering and accessing post-abortion care in Kilifi County, Kenya. The data collection approach included participant observation and informal conversations in public health facilities and neighboring communities, as well as in-depth interviews with 21 girls and young women treated for abortion complication and 12 healthcare providers.

**Results:**

Our findings show that almost all patients described the MVA as the most painful procedure they have ever experienced. The unbearable pain was explained by various factors, including the lack of preparedness of health facilities to offer PAC services (i.e. lack of pain medicine, lack of training, inadequate knowledge and grasp of pain medication guidelines, and malfunctioning MVA kits). Moreover, the attitudes of healthcare providers and facilities management toward the MVA device limited the supply and replacement of MVA kits. Moreover, the scarcity of pain medicines also gave some providers the opportunity to abuse patients guided by their values, whereby they would deny patients pain medication as a form of "punishment" if they were suspected of inducing their abortion, especially adolescent girls.

**Conclusion:**

The study findings suggest the need for clearer guidelines on pain medication, value clarification and attitude transformation training for providers, systematizing the use of medical uterine evacuation using medical abortion drug and strengthening the supply chain of pain medication and MVA kits to reduce the pain and improve the quality of post-abortion care.

## Introduction

Unintended pregnancies can be stressful, especially with the restrictive laws around termination of pregnancies. Yet, sub-Saharan Africa (SSA) is one of the regions with the highest unintended pregnancy rates and the strictest abortion laws [1]. Although 21 countries in SSA have changed or introduced laws to expand the legal grounds for abortion since the 2000, evidence shows that 92% of women of reproductive age in the region live under highly or moderately restrictive abortion laws [2]. In Kenya for instance, abortion is only allowed to save the life or health of the mother, or if permitted by any other written law [3].

Globally, 60% of unintended pregnancies end in induced abortion, of which the majority are unsafe [1]. In Kenya, approximately 500,000 induced abortions occurred in 2012, of which majority were unsafe and led to complications and deaths [4, 5].

For these reasons, post-abortion care (PAC) was introduced in the health policies under the umbrella of “saving lives” since the cases were alarming. PAC can be implemented in any country no matter how prohibitive the laws against abortion, as it deals with treating a woman after induced or spontaneous abortion [5]. In line with the government commitments, Kenya has developed guidelines in 2012 to prevent death from unsafe abortion, which were then withdrawn [6] and post-abortion care guidelines in 2019 to direct providers on the different components of PAC [7].

Over the years, PAC services delivery has focused on key elements including treatment of complications such as incomplete abortion [8]. The methods for treating incomplete abortion have evolved over time, from surgical methods such as dilatation and curettage (D&C) to vacuum aspiration both electrical and manual, and medical abortion (MA) using Misoprostol [8, 9]. The Kenyan PAC guidelines recommend both MA and Manual vacuum aspiration (MVA) [7].

While MA is gradually gaining traction in abortion and PAC spaces, MVA has been a commonly used procedure to treat incomplete abortions since it is deemed safer, less costly and effective compared to D&C that requires significant resources and manpower (i.e. general anesthesia, theater, input of a medical doctor, and hospitalization) [9, 10]. The benefits of MVA include task shifting in the management of PAC from doctors to nurses, decentralization of services from high-level to low-level facilities as well as reducing the number of hospital admissions unlike D&C [10–12]. Although MA is being more and more recommended for its effectiveness and low cost for the health systems [13], MVA continues to be given priority in countries such as Kenya. However, this technology comes with deep intense pain and lower abdominal pain that need to be addressed beforehand to ensure quality care [14]. Pain medication has shown to be effective in preventing painful MVA procedures. However, while pain management is key in ensuring patient-centered post abortion care, studies show that women are often treated with no pain control. During the initial introduction of the procedure in Kenya, a study documented that 55% of women who underwent MVA procedures to treat incomplete abortions experienced pain and only 19% had received pain medication [15]. Recent studies continue to highlight instances where women are treated without pain medication in Kenya [16, 17]. Among other factors contributing to lack of attention towards pain control during MVA are stigma towards abortion, reasoning that pain medication is unnecessary and the lack of clarity on pain management guidelines [12].

Building on the previous evidence on MVA pain and factors driving it, this paper explores the lived experiences of pain during the MVA processes as observed and as reported by patients, while also focusing on health care providers’ practices in the management of pain during the procedure in Kilifi County, Kenya.

## Methods

### Study design, setting and population

This paper is drawn from a larger ethnographic study conducted in Kilifi County between January and July 2021. The study aimed at understanding girls and young women abortion decision-making process, their care seeking pathways, as well as the different actors involved. The 2014 Kenya Demographic and Health Survey indicates that almost two out of ten girls in the Kilifi between the ages of 15 and 19, are reported to be pregnant for the first time or have had a child. This places Kilifi among the counties with a high prevalence rate of teenage pregnancy (21.8%). One of the reasons for this high teenage pregnancy rate is the low rate of 33% in usage of modern contraception in the county, against a national rate of 53% [18]. Consequently, unsafe abortions are reported to be common in the county, especially among 15-19-year-old girls, with some ending in complications treated in public health facilities. The study was conducted in two of the nine sub-counties: one urban (Kilifi North), and one rural (Kaloleni). In these two sub-counties, we collected data in two primary and two referral level facilities, as well as the neighboring communities. While the main study targeted various profiles of participants (including girls and young women who had abortion, community members, services providers and policy makers), this paper focuses on girls and young women who received post-abortion care following abortion complications as well as health care providers in charge of PAC services provision.

### Data collection approach and participants’ characteristics

The data collection involved participant observation in the targeted public health facilities and surrounding communities, as well as in depth interviews with girls and young women who have experienced abortion and health care providers offering post-abortion care. Four junior researchers with backgrounds in anthropology and sociology were taken through face-to-face training for five days. The training included the study objectives and design, human research ethics, ethnographic approach, and interviewing and notes taking techniques. To check the validity and reliability of the study tools and documents, researchers did pretests and role-plays, and the tools refined accordingly.

After the training, the researchers immersed themselves in the targeted facilities and communities for six months. The researchers, first, liaised with the facility in-charges who showed and introduced them to specific wards and individuals in charge of providing PAC services. During each typical day of observation in the facilities, the researchers would assist the staff with entering records, registering patients and helping them to navigate the facility. Whenever a PAC patient was admitted, they would seek informed consent from the patient and follow the treatment procedure. For MVA procedures, they assisted by comforting and encouraging the patient, or holding a flashlight to enable the healthcare provider to clearly see the cervix and perform the procedure. This helped them to observe and document patients’ interactions with providers while engaging them in an informal conversation. Not only did this help them build rapport with service providers but also with the patients.

After observing the care process, researchers then asked consent from the patients (who were between 15 and 24 years) to be followed up and interviewed at a later point. In total, 16 girls and young women agreed and were followed up in their communities for six months. This involved visiting them at their home, accompanying them in their daily activities such as managing their shops, fetching water, or mentoring some girls and their family members. In addition to helping us understand their living conditions and factors that might have contributed to their vulnerability towards unintended pregnancy and unsafe abortion, it helped in building rapport with the participants who then could freely speak about their experiences. In the process of following up on these girls and young women, we also identified, through community health volunteers and youth advocates for reproductive health, five other girls and young women who had had abortions (including PAC), who were also recruited for the study and regularly followed up. This brought the total number of participants to 21. In addition to following them by observing their daily lives and informal conversations, we also conducted repeat in-depth interviews to get deeper understanding of their abortion and post abortion care experiences. The interviews and discussions were conducted in Swahili, and sometimes in English. The researchers also took field notes during these informal conversations with either health care providers or the PAC patients.

The immersion in the primary and public health facilities helped us to identify 12 health care providers offering MVA, and conduct in-depth interviews with them. The providers included seven clinical officers, four nurses and one medical doctor. The interviews and conversation with providers during the observations focused on understanding their experiences providing PAC, their perceptions of these services, and the challenges faced.

### Data management and analysis

Formal interviews were audio-recorded after obtaining consent from the participants. Audio files and field notes were anonymized, and stored in a password-protected Google Drive project folder. All researchers installed two-step verification to access the data. The audio files were then transcribed verbatim and translated into English when needed. The data was analyzed using a thematic analysis approach [19] driven by theory and data. The study team and research assistants first went through both the study protocol and a sample of interview transcripts and observation notes to draw the emerging themes and sub-themes, and create the codebook. The majors themes in the codebook for this paper included experiences of pain, supplies and equipment, skills and staff training on PAC, records of PAC cases, informal services provision, values-beliefs-attitudes and services provision, patient-provider interactions (induced versus spontaneous, young versus adult, married versus single). The coding was done by three members of the team using Dedoose software. Inter-coder reliability test was first performed to ensure that every coder has the same understanding of the codes and to capture emerging codes. After that was achieved, the coders proceeded to code independently. Online weekly meetings were scheduled to discuss memos, emerging themes or duplication as well as get clarifications on unclear codes. Findings from interviews with participants and observation notes were presented through verbatim quotes adhering to the protection of the participants’ identities.

### Ethical considerations

The study protocol was reviewed by the African Population and Health Research Center (APHRC) internal review committee to ensure scientific soundness (DOR/2020/036). We also sought and obtained ethical approval from the Amref Health Africa’s Ethics and Scientific Review Committee (ESRC) (AMREF-ESRC P909/2020). A research clearance was also obtained from the National Commission for Science Technology and Innovation (NACOSTI) (License No: NACOSTI/P/21/8421). As part of community entry, key stakeholders such as the local administrative and health authorities, County Commissioner, County Directors of Education and Health, health facility administrators, chiefs, assistant chiefs, and village elders were engaged first to seek their support. They were then introduced to the research assistants.

The study participants were consented and confidentiality of collected information was also provided. Given the sensitivity of the issue of abortion, we requested and obtained waiver for written consent for participants who did not want to have their consent documented. Therefore, the consents were either written or oral. The participants were duly notified that the decision to participate or not to participate was voluntary, and they had the freedom to withdraw from the research if they wished to do so without coercion. For participants below 18 years, we did not seek parental consent due to the stigma and covert practice around abortion. In most cases, parents and guardians were not aware of the abortion, hence seeking consent from them would put girls at risk of stigma or abuse. Based on this reasoning, we requested and obtained a waiver of parental consent from the ethics committees.

## Results

The woman is crying in a lot of pain, the doctor says *“sorry mama*, *sorry*, *try to cough*, *it will end soon”* …He says he should have given the woman painkillers …*“This woman is in pain*, *she needs painkillers*’’. When the suction is about to end, he picks the small tube to check if there is no tissue, then the suction tube gets full. “*Just when we thought we were done*, *here is more…*.”

(Observation notes, referral level facility, urban setting)

These field notes highlight the pain women and girls experienced while we were observing MVA procedures in the health facilities. It sheds light on how the pain is lived by the patients and the providers, as well as the factors that could explain such pain. Table 1 summarizes the socio-demographic characteristic of the patients observed and/or interviewed in this study.

**Table 1:** Sociodemographic characteristics of adolescents and young women interviewed.

Characteristics		Frequency (N = 21)
**Age (years)**	14–17	4
	18–19	5
	20–24	10
	25–30	2
**Education level**	Primary school	7
	High school	11
	College	3
**Area of resident**	Urban	6
	Peri-urban	7
	Rural	8
**Marital Status**	Married/cohabiting	2
	Separated	1
	Never married	18
**Occupation**	Student	9
	Employed/informal laborer*	8
	Unemployed/housewife	4

*hairdressers, house girls, bartender, shopkeepers, waitress

### When the cure hurts

Nearly all the girls and women who were treated using the MVA procedure narrated their experiences as ‘*very painful*’ as highlighted in the following quote:

*I was seeing that I*’*m going to just die*. *I was feeling a lot of pain because there is no numbing injection*. *You feel the thing being pulled*. *That is*, *you feel it completely*. *It is not like giving birth at all*. *Giving birth is easier*.

(18 years old, single, unemployed)

As described in the quotes and the field notes above, we witnessed patients going through such pain and equating it to the feeling of someone *“inserting their hands through the vagina forcefully”*, or *“pulling out something”*, hence making it unbearable. Patients who already experienced childbirth found the MVA pain worse than that of delivery. To express their pain, some women cried, shouted, screamed while others tried as much as they could to restrain themselves from shouting or letting out a sound that could alarm people outside the room. They would then moan and fidget silently, close their eyes tightly, let the tears flow down to their hair, bite their teeth together, sweat, sway their hips while squeezing their hands. When the pain became intense, they requested to hold our hands. Through the discussion and interviews, the participants explained that stigma around abortion affected the way they reacted to pain during MVA. Given the stigma surrounding abortion in their communities, some women and girls would avoid seeking post-abortion care in the facilities to avoid providers’ reactions or meeting members of their community in the facilities. Those who were brave enough to seek care explained that they needed to do it discreetly, which included bearing the pain in silence not to draw attention to their case. To echo this, a 32-year-old woman reported that she restrained from shouting for fear of being heard and let her secret out as highlighted in the following quote:

*It was painful*, *but I could not remove the sound…you saw there was a window and the window was open*, *and there were people out there*. *If I could be noisy*, *people would start to ask and wonder what is going on there*, *and then they would follow up my case*. *So*, *I just had to endure the pain*.

(32 years old, separated, casual worker)

Aside from stigma, some women and girls felt they had to endure the pain in exchange for PAC services. One of the women mentioned that she knew the provider should provide pain medication but chose not to ask for fear of how the provider might react. Moreover, some patients had to suppress the pain as much as they could because they believed the provider might be mad at them to an extent of not completing the MVA procedure as it happened in some cases where patients could not endure the pain, as highlighted in the following notes:

Mary (pseudonym) is crying in pain whenever the suction tube is inserted *“Don*’*t do that*, *I don*’*t want*, *do it slowly”*, she cries. …Mary keeps crying, putting her hand close to the vaginal opening. *“Don*’*t do that*, *you will get a very bad infection”* the doctor says. The doctor tries to insert another tube for the suction, Mary cries *“I don*’*t want any more of this*, *it is painful*. *I want to go home*, *I want to go home*!*”*, as she is trying to close her legs. At this point, the doctor has stopped the vacuum suction. She lies there, Dr. G says, *“you can discharge her against doctor*’*s advice*, *we can*’*t force her if she doesn*’*t want*.

(Observation notes, referral level facility, urban setting)

That day, the providers decided not to continue the procedure and termed the patient as “non-compliant”. Facility management and the girl’s mother had to intervene for the procedure to be completed late in the evening.

Unlike these providers, others would stop the procedure, and engage the patients in friendly chat with the aim of drifting their thoughts away from the pain. When the patients exhibited pain, they would tell them “*sorry mama*, *sorry*, *try to cough*, *it will end soon*”, “*be strong*, *we are almost done”*. With such constant engagement, they were able to complete the procedure. In situations where this moral support happened to be insufficient, they would resort to pain medication (when available), or request help from colleagues to hold the patient in order to complete the procedure.

We noticed that whenever they fidgeted, moved their hips, held the doctor’s hands or shouted, they interfered with the procedure, which ended up taking longer, hence prolonging the pain. Such MVA procedures would therefore last up to one hour instead of 15–20 minutes for procedures where the woman bears the pain and stays calm.

### Factors driving the pain: Between lack of preparedness and providers attitudes

Nurse B began preparing to perform the MVA and he gathered the MVA kit that was in the sterilized solution. He started assembling it and he asked me to help him as he told me about the different cannulas and he noted that the white and yellow ones were missing. I put on gloves, he asked me to give him a condom and that was the first time I saw him using a condom during an MVA. He placed it inside the sanctioning part and he told me it helps make the work easier because it reduces the friction when opening it and closing it when pumping. He asked me to pass the lubricant, he applied it to the speculum and inserted it in the patient. He then told the patient *“I*’*m sorry this is going to be painful”* and then he inserted the cannula…

(Observation notes, referral level facility, urban setting).

These observation notes show that the provider did not use pain medication before starting the process as recommended. Hence, he knew the pain the patient was likely to go through, and went ahead to prepare her to expect it. In addition, the MVA kit was worn out, forcing him to use a condom to ensure the MVA draws properly. Overall, the study pointed out various factors driving the painful MVA procedures that we group into two categories: health system related factors (i.e. malfunctioning MVA kits, lack of training, lack of pain medication drugs) and provider related factors. Altogether, this led to pain medication not being used, or pain medication being used but ending up to be ineffective as illustrated in the field notes.

### Lack of preparedness of health facilities to offer PAC

#### Challenges on the availability and use of pain medication

As illustrated in the field notes above and in many other cases, women were not given painkillers before the MVA was performed. This was explained by the lack of pain medication due to recurrent stock out. The situation was worse in rural facilities where MVA were often done without pain medication because providers did not have the pain drugs in stock, as compared to urban facilities where providers reported a greater availability of pain medication, but not being used for other reasons (we will describe those reasons later). While we were observing at the health facilities, we often heard nurses, midwives and doctors complaining about things that they missed, yet “crucial” for service provision:

*I don*’*t know what is happening at the store and pharmacy*, *we are missing crucial things*, *and I don*’*t know how they want us to work*.

(Health care provider, referral level facility, peri-urban setting)

This included crucial equipment and supplies such as sterilized gauze, MVA kits, and medicines to handle patients (including PAC patients). Therefore, providers could not use them before performing the MVA and would prescribe oral painkillers to patients after the procedure was completed.

When the pain medications were available and providers could use them, our data also show a lack of clear understanding of the pain medication to be used, hence leading to the use of various types of pain medication and different dosages. Among the medicine cited were Tramadol, Lidocaine and Panadol. Moreover, some providers thought that giving painkillers to the patients would impede the uterine evacuation process as they also use other medicine such Oxytocin to increase the uterus contraction to expel the conception product:

*Ever since I started being a nurse*, *I have never given them painkillers*. *We just do the procedure*, *but yes*, *we assess the condition*. *But like her case*, *the pregnancy was already out*. *So*. *if we give her painkillers*, *the Oxytocin won*’*t work since it is supposed to help in contracting the uterus*. *The contractions are painful*.

(Health care provider, primary level facility, rural setting)

In some cases, providers would give patients an injection before the beginning of the procedure, and when confronted with heavy pain (or lengthy procedures because of malfunctioning kits), they would inject another dosage before continuing with the procedure.

#### Faulty and malfunctioning MVA kits

Another major challenge that providers faced during MVA procedure was faulty or missing MVA instruments. As a result, the procedures took longer and exposed patients to prolonged pain. During one of the procedures observed in a referral facility, the procedure took close to one hour and the patient was screaming in pain. The provider in charge of the procedure kept requesting for different cannulas (the green one, then the blue one, etc.) while complaining that the MVA kit had worn out since it was not creating the vacuum required. In similar cases, we witnessed providers’ frustrations when faced with malfunctioning MVA kits and could hear them vent about missing cannulas, broken kits or worn out kits as highlighted below:

*This vacuum is not sucking*, *it is not producing that sound*…*I wish I had brought my kit*, *this cannula is not working well*.

(Health care provider, referral level facility, urban area)

From the discussions with providers, MVA kits are meant for a certain number of procedures (i.e. 25 times for some of the IPAS products), after which they become obsolete. However, providers explained that they have been using some of these materials for months. For referral level facilities, this implied many procedures performed, since they could not spend one week without performing at least one procedure. This resulted in kits warning out, breaking or not sucking.

Providers reported that they make request for replacement of MVA kits, but the process always takes long because facilities management accuses providers of “inappropriately” using MVA kits, as explained by one provider:

*You know I don*’*t understand the administration*, *every time MVA kit spoils they take forever to replace*. *They are always complaining that the people at the casualty are careless*. *They think it is supposed to last forever*. *They also say that it is misused to perform criminal abortion*. *So*, *they think that by not replacing them they are punishing us*, *which in reality they are not*. *When patients come requiring MVA I tell them to go elsewhere because what else can I do*?

(Health care provider, referral level facility, urban area)

As highlighted in the quotes, the replacement or supply of MVA kits is trapped between providers and health facilities management perceptions of the equipment: used to provide PAC or to induce abortion. While discussing with facility management on supply, they further justified the delay on replacing MVA kits by the lack of evidence on what the kits are used for. They explained receiving recurrent requests for kits replacement, yet they do not have evidence on the number of cases treated in the facilities. This according to them is due to providers using the MVA kit unofficially and not recording cases, which makes them *“not see the need to supply when there is no evidence on provision of PAC in the records”* (facility management staff, referral level facility). Indeed, we also observed practices around such privatization of the MVA services through patients reporting that they paid money directly to providers yet PAC services are deemed free in level three and four facilities in the county. We also encountered situations where we checked the register when we arrived in the morning and did not find any PAC cases, and later on got to hear about patients that were treated during the night from providers who handled them without registering.

To avoid situation where MVA kits would be broken and taking long to be replaced (as result of providers using it to induce abortion or to provide PAC and getting money for themselves), some unit managers found strategies to ensure the MVA kits last longer. This involved hiding or locking the MVA kit or cannulas in their office when off duty to ensure they have control over who uses it, as reported by one provider:

*By the way*, *I just learnt that the MVA kit is missing*, *like it vanished*. *But K* (name of a provider) *has told me he thinks P hid it*, *you know he doesn*’*t like us doing the procedure*. *He also always hid the small yellow cannula*

(Health care provider, referral level facility, urban area)

Though unit managers did not comment on that, we noticed that the MVA kits were not available during nights or weekends, which coincided with times where those managers were absent. The implication of such practices was some patients having to wait for long before receiving care, hence going through prolonged pain and risking their condition worsening, as well as some being referred to other facilities.

To overcome the challenges of malfunctioning MVA kits, some providers used their own kits (or borrowed that of colleagues), while others would improvise. Using a condom to create a vacuum was one of the strategies used by providers to perform procedures in cases where the vacuum was faulty or worn out, as described in the field notes above. Such strategies could explain that some patients still feel a lot of pain even in cases where they were given pain medication before the beginning of the procedure.

Our findings also show that some providers did not know how to properly use the MVA kits as captured in the observation notes below:

The clinical officers (they were two) start preparing to do the MVA… They then start consulting each other on what to do. *“This vacuum is not sucking”*, one of them says. Meanwhile the patient is in pain, yet they had injected her with painkillers. She bites her teeth together, and she is sweating profusely. After several tries without success, I think of telling the clinical officers on what they might be doing wrong, having observed other MVAs, I feel like I have seen how the vacuum works, since the other doctors were also taught in my presence, but I also want to observe at what point they will call for assistance, or what decision they make. So at this point, since I see the pain the patient is going through, I feel I can give a small hint. They had not created a vacuum because they were not using the buttons that create the vacuum. One of the clinical officers says, *“this thing is not working*, *it*’*s not producing that sound”*, then I suggest *“what about the buttons*?*”* just to give them a hint of something they might remember

(Observation notes, referral level facility, urban setting)

Because they were interns or did not receive official training on how to perform MVA, we observed some providers fumbling from assembling MVA equipment to performing the procedure, leading to lengthy and painful procedures. Lack of training on PAC also affected providers’ ability to understand the rationale for providing pain medication, which pain medication to give and how to give it. During our presence in the field, one of the providers went for a training on PAC and learnt about pain management during MVA for the first time, which she termed as *“MVA without pain”*. Before then, she explains that she did not know that MVA could be done without pain as she stopped giving pain medication before the procedure after she concluded that it was not “useful” since patients would still experience pain. But during that training, she learnt about “Paracervical block”. This is an anesthetic procedure used in obstetrics, which consists of injecting some Lidocaine to the cervix to make the patient numb to the pain:

*I have been taught to give a Paracervical block and then perform the procedure*. *You can give an oral drug like Brufen before and give the Para cervical block*. *It has sharpened my skills*

(Health care provider, referral level facility, urban setting)

Since the training, she has been implementing this approach and noticed reduction in patients’ complaints about pain.

#### MVA pain a punishment tool?

While some providers did not use medicine because they did not know how to use it or because the pain medications were not available, some providers deliberately chose not to use pain medication for various other reasons. When asked about his practice regarding pain medication during MVA, one of our participant responded:

*We don*’*t usually inject pain medicines*, *it depends on the situation because you will find some are in so much pain so you inject them but for the small small ones you just do the procedure; they aren*’*t in pain*.

(Health care provider, primary level facility, rural setting)

Like this provider, others also reported that they give pain medication based on their subjective assessment of the level of pain the patient is going through before the procedure, including whether she is screaming or not. Yet, some patients were already going through pain but did not express it, hence, providers would categorize them as “small small” pain. Our findings also show that some providers, whether the patient was in pain or not, did not use pain medication because the medication was reserved for “genuine cases”, which included women with spontaneous abortion or older women. Most providers observed and those interviewed reported that they were against abortion considered as a sin. As such, girls and women who induced their abortion and face complications would be treated with disdain as the following:

*That girl came here last night she is stupid*. *Instead of her telling the truth so that we would help her*, *she kept lying till later when Sister had refused to attend to her*. *That*’*s when she said that she had been given some abortive medication by her boyfriend*. *I don*’*t know what is wrong with todays*’ *generation*.

(Health care provider, primary level facility, rural setting)

During the care process, providers would “grill” patients, take them through a long questioning to make them “tell the truth” before they would “help” them (as highlighted above). In situations where patients refuse to accept that they induced the abortion, some providers would rely on the age and marital status of a patient to make their diagnosis. Young and unmarried girls were often suspected of inducing their abortion, compared to older and married women:

*We look at the age and their marital status*. *Many of these people you will just see when they come in*. *The younger ones will say they are just bleeding*. *They come in panicking because they see they are bleeding and they have done something at home*. *So you can see when someone is bleeding and panicking*. *Many times when you find a grown woman with a husband and other children*, *which is most likely a spontaneous abortion*.

(Health care provider, referral level facility, urban setting).

When offering MVA services to patients suspected of inducing her abortion, providers would punish them by subjecting them to MVA pain to ensure they do not terminate a pregnancy again. Providers explained that since they did not have enough medication, they needed to prioritize cases. Hence, they would choose to give the pain drug to “genuine cases”, who were mainly patients who did not cause their condition, i.e., inducing their abortion and facing complications. On the other hand, patients who terminated their pregnancy were perceived as being the main cause of their bad health condition, therefore did not deserve empathy. Those patients were expected to feel all the pain, which according to providers, would teach them lessons not to repeat again, and also make them share their experience with other girls in the community to prevent. One of the providers was laughing while informing the patient ahead of the pain she was to expect:

*Do you know any traditional songs*? *Today you will sing even the ones you don*’*t know*, *because it will be very painful (laughter)*.

(Health care provider, primary level facility, rural setting)

While such “non genuine” cases were going through the pain and failing to remain calm, they would be scrolled at, verbally abused by providers:

*You need to open your legs widely like when you were having sex*. *It was sweet then now you will have to endure the pain*.

(Observation notes, public facility, rural area)

*Cooperate here like when you were cooperating during sex*, *this is the implication of the sweet sex you had*.

(Observation notes, public facility, urban area)

Some providers would also threaten to slap the patient or leave the procedure halfway if the patients continued to fidget or scream in pain, as explained by one provider:

*I usually tell my patients to endure the pain so that we can finish it all at once*. *If they scream and disturb me*, *then I leave them with the bleeding*. *The pain will still be there and they might even get sepsis and die*. *I normally tell them the truth*.

(Health care provider notes, public facility, urban area)

When told this in a hush tone, patients would try to hold themselves to ensure the procedure is fully completed, regardless of how much pain they were in.

## Discussion

This study provides insights into how women facing incomplete abortion experience MVA pain, as well as the factors driving such pain. Although the Kenyan post-abortion care guidelines recommend both MA and MVA as procedures for treating incomplete abortion, especially in the absence of severe vaginal bleeding, our study found that most providers in the study facilities had a preference for MVA for various reasons, including convenience of the procedure for them, financial reasons, and the unavailability of MA medicine in facilities stocks. When performing the MVA, most providers did not use the pain medication or when they used it, it failed to prevent the patients from experiencing pain because of lengthy procedures. Factors explaining the non-use of pain medication and painful procedures despite pain medications being used were the lack of preparedness of the facilities to offer PAC services, namely the lack of medication, lack of training, poor knowledge and understanding of the pain medication guidelines, as well as malfunctioning equipment. While different studies pointed out the lack of preparedness of public health facilities in sub-Saharan Africa and in Kenya more specifically [20–23], these findings show how the lack of preparedness is lived by providers and patients and how it affects the quality of care. This lack of preparedness translated into preference of MVA using faulty devices, hence rendering procedures painful, instead of medical uterine evacuation (MA) shown as equally effective [13, 24–26]. In addition to its effectiveness, studies in Kenya and other countries have described MA as safe(r) (compared to MVA were risks of perforation and infections exist), cheaper, highly accepted by patients and adapted for task shifting in the health system [17, 25, 27, 28]. Studies also showed how it decreases the burden on the health system, especially for limited resources countries such as Kenya and contributes to increasing access to PAC services [13].

Moreover, the supply and availability of the MVA kits is further affected by health care providers and facility management staff’s perceptions of abortion (“real” PAC cases versus induced abortion) and the privatization of PAC services. Hence, this makes PAC cases invisible in facilities records, and subsequently affect timely supply of equipment and quality of the services, as also documented in other countries [29–31].

The lack of preparedness and the scarcity of PAC resources in health facilities also gave providers the opportunity to abuse patients guided by personal values that oppose abortion and perceptions of MVA as a tool. These findings build up on evidences that discussed how perceptions of reproductive technologies such as MVA could drive individual attitudes and behaviors towards its use [29]. As described in other settings, providers feel discomfort and frustrated when offering PAC to women who induced their abortion [32]. To overcome such feelings, using MVA instead of MA, or not providing pain medication (as also documented in this study) appears as a solution [32]. Providers did not use pain medication, because they felt patients who induced their abortion needed to be punished for their sin [12]. While all patients suspected of inducing their abortion could potentially be denied pain medication, our findings shows that adolescent girls were more likely to face this situation compared to older and married PAC patients. Yet, evidence shows that younger women are likely to experience more pain compared to older patients when not given pain medication for biological reasons. A study conducted in Uganda found that the odds of intra-procedural pain decreased with increasing age of the women, with teenagers (age<20 years) experiencing 8 times higher odds, while women aged 20–24 years have 4 times higher odds, and those aged 25–30 years 3 times higher odds of intra-procedural pain [33]. This difference in pain experiences was explained by changes the nervous system that lead to a reduced pain perception with age (28). Therefore, there was a need for more pain control measures among such group.

Yet, because of the stigma, they were always likely to be treated without pain medication, hence subjecting patients to obstetric violence, which has been conceptualized as “harm inflicted during or in relation to pregnancy, childbearing, and the post-partum period” [34]. Evidence shows that obstetric violence can be both interpersonal and structural: it can manifest through providers/patients interactions with providers acting as perpetrators and also occur from broader political and economic arrangements that disproportionately harm marginalized populations [34]. In our case, providers often deliberately did not use pain medication when attending to women suspected to have induced their abortion, and chose to keep painkillers for “genuine cases”. Such behaviors were enabled by structural factors such as abortion stigma, lack of preparedness of health facilities to offer PAC, and the confusion and vagueness created by the withdrawal of the standard and guidelines for reducing morbidity and mortality from unsafe abortion by the Kenyan government. The findings also showed how single and young women are more likely to face such obstetric violence. Literature has indicated that these young women particularly find themselves in vulnerable situations when it comes to abortion stigma because of the taboo around their sexuality [35, 36]. Yet, subjecting women and girls to such pain can affect their mental health. Evidence show that when women go through pain while undergoing reproductive procedures it can lead to posttraumatic stress [37]. This in turn can impact their health outcomes such as adherence to post-abortion guidance (refusal to return for follow up visits) and their future health seeking decisions [17].

### Limitations and strengths of the study

This study is one of the rare in sub-Saharan Africa that explores the lived experiences of the MVA pain during post-abortion care. The ethnographic approach used (through the participant observation for instance), allowed us to capture the emotions, feelings, as they were happening, as well get a deeper understanding of the factors driving the pain. However, as a qualitative study, the findings may not be generalizable to other settings. In addition, given the fact that the focus was only on public health facilities, the study findings do not inform on experiences and practices around pain in private health facilities.

## Conclusions

This study has provided important data on how the lack of clear guidelines, limited resources and training as well as providers’ conceptions of PAC technologies, turns a procedure deemed to improve PAC quality into medical and obstetric violence. Such poor experiences affect women’s health seeking behavior when faced with similar conditions. These findings suggest the need for clearer and better disseminated guidelines on pain medication, value clarification and attitude transformation training for providers, and strengthening the supply chain of pain medication and MVA kits to reduce pain and improve the quality of PAC. These findings point out also the need for systematizing the use of MA through the training of providers and supply of health facilities. Ultimately, this will help girls and young women to make timely decisions to seek PAC in health facilities when faced with abortion complications, and become ambassadors for safe abortion in Kenya and low and middle-income countries.
